# Rifampicin-resistant TB in paediatrics: a patient series from a secondary health facility in Northern Malawi

**DOI:** 10.5588/pha.25.0055

**Published:** 2026-05-18

**Authors:** S. Chitsulo, D. Omotayo, J. Furin, Y. Saidi, M. Chisale, B. Mbakaya, F. Sinyiza, T. Mwenyenkulu

**Affiliations:** 1 Ministry of Health, Karonga District Hospital, Karonga, Malawi;; 2 Ministry of Health, National TB Program, Lilongwe, Malawi;; 3 Department of Global Health and Social Medicine, Harvard Medical School, Boston, MA, USA;; 4 Mzuzu University, Mzuzu, Malawi;; 5 Livingstonia University, Livingstonia, Malawi;; 6 Mzuzu Central Hospital, Mzuzu, Malawi.

**Keywords:** tuberculosis, child TB, drug-resistant TB, RR-TB

## Abstract

Childhood pulmonary TB diagnosis remains a challenge due to difficulties in obtaining suitable specimens and the paucibacillary nature of the disease. Recent advances in sample collection and diagnostics could improve care for children, especially those with rifampicin-resistant TB (RR-TB). Although these have been recommended by the WHO, there have been limited reporting of the clinical impact of these novel approaches. We report the clinical impact of stool sampling in four children at Karonga District Hospital in Malawi. All four children were diagnosed with RR-TB, received all-oral regimens, and experienced successful treatment outcomes.

Rifampicin-resistant TB (RR-TB) is a form of drug-resistant TB, which is resistant to rifampicin. It is a serious threat to the public and in children is mostly acquired through close contact with an infectious person.^1^ Diagnosis remains a challenge due to difficulties in obtaining suitable specimens from children and the paucibacillary nature of the disease.^2^ Globally, an estimated 1.1 million children develop TB annually, yet only 37% are diagnosed and treated.^3,4^ Modelling estimates suggest that between 25,000 and 33,000 children become sick with RR-TB each year, and only few are diagnosed or started on treatment.^5^ The World Health Organization (WHO) has recently recommended several strategies for improving TB and RR-TB diagnosis in children. These include collection of samples such as stool and the use of more sensitive cartridge-based nucleic acid amplification tests such as Xpert MTB/Ultra.^6,7^ While there have been multiple studies validating these approaches to the diagnosis of paediatric TB, there have been limited reports on how these tools impact the clinical care in high-burden settings.^8^ RR-TB treatment is complex, but newer, all-oral regimens with drugs like bedaquiline and delamanid are improving treatment outcomes and associated with lower rates of toxicity.^9^ Malawi, a TB/HIV high-burden country and childhood TB accounts for 9% of all incident cases.^10,11^ We will successfully report four patients of RR-TB in children.

## METHODS

Patient’s reports were retrospectively presented based on the records at the facility from 2023. Upon presentation, history taking, physical examination, and investigations like chest X-ray (CXR) and laboratory tests were done (Table). Formal informed consents were taken from the patients’ guardians and all identifying information has been removed to protect the privacy of the patients. All patients had their stool processed using the simple one-step method and tested using Xpert MTB/RIF Ultra (Sunnyvale, CA, USA).^12^

**TABLE.** Baseline assessment tests and results.

Tests	Patient 1	Patient 2	Patient 3	Patient 4
Weight (kg)	28.9	10.5	16.6	10.6
Height (cm)	139.0	75.0	103.0	104.0
MUAC (cm)	15.5	13.5	13.0	12.5
Hb (g/dL)	12.6	9.8	10.1	11.2
WBC (10^3^/µL)	5.0	9.2	11.3	6.4
Platelets(10^3^/µL)	243	461	385	264
ALT (U/L)	13.2	4.60	13.7	11.6
AST (U/L)	28.2	40.84	26.1	ND
ECG (QTcF)	426	370	390	409
Albumin (g/L)	4.35	4.26	4.33	4.13
Creatinine (mol/L)	0.14	0.47	0.73	0.56
Eye examination	R6/6 L6/6	R6/6 L6/6	R6/6 L6/6	R6/6 L6/6

## PATIENT SERIES

### Patient 1

**FIGURE 1.**
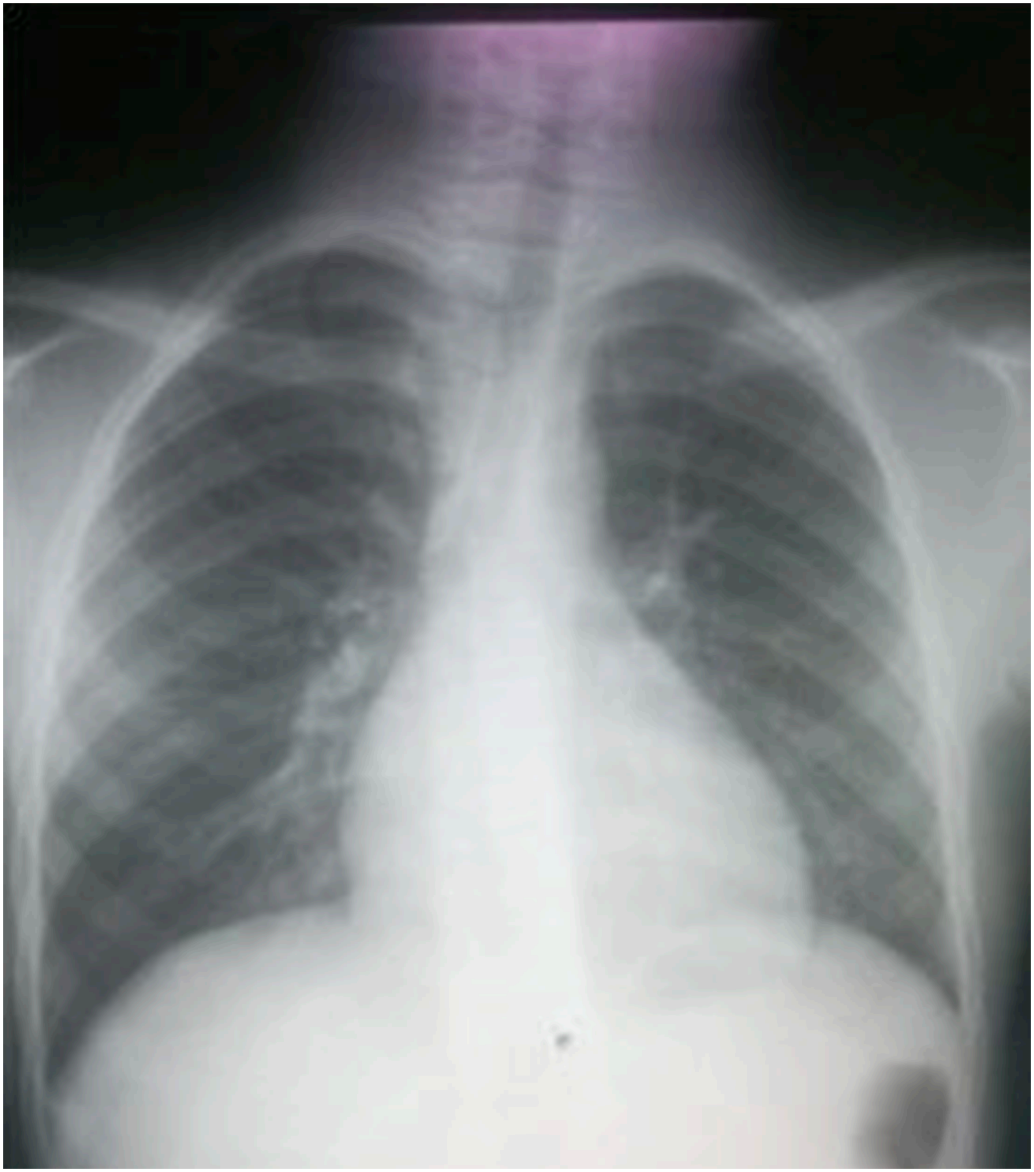
Chest X-ray of patient 1, which had right radio sternal lymph node enlargement.

A 9-year-old boy presented with history of neck swelling, weight loss, and reduced playfulness. Eight weeks prior to the current presentation, he had cough which was treated with Amoxicillin. No family history of TB was reported. He had cervical lymph nodes which were painless and asymmetrical. CXR showed radio sternal lymph node enlargement pushing the trachea to the left (Figure 1). Stool sample was collected, which came MTB detected high and RR. Nine-month regimen was initiated with bedaquiline (2Bdq-Lfx-Lzd-Cfz-Cs/4Bdq-Lfx-Cfx-Cs/3Lfx-Cfx-Cs).

### Patient 2

**FIGURE 2.**
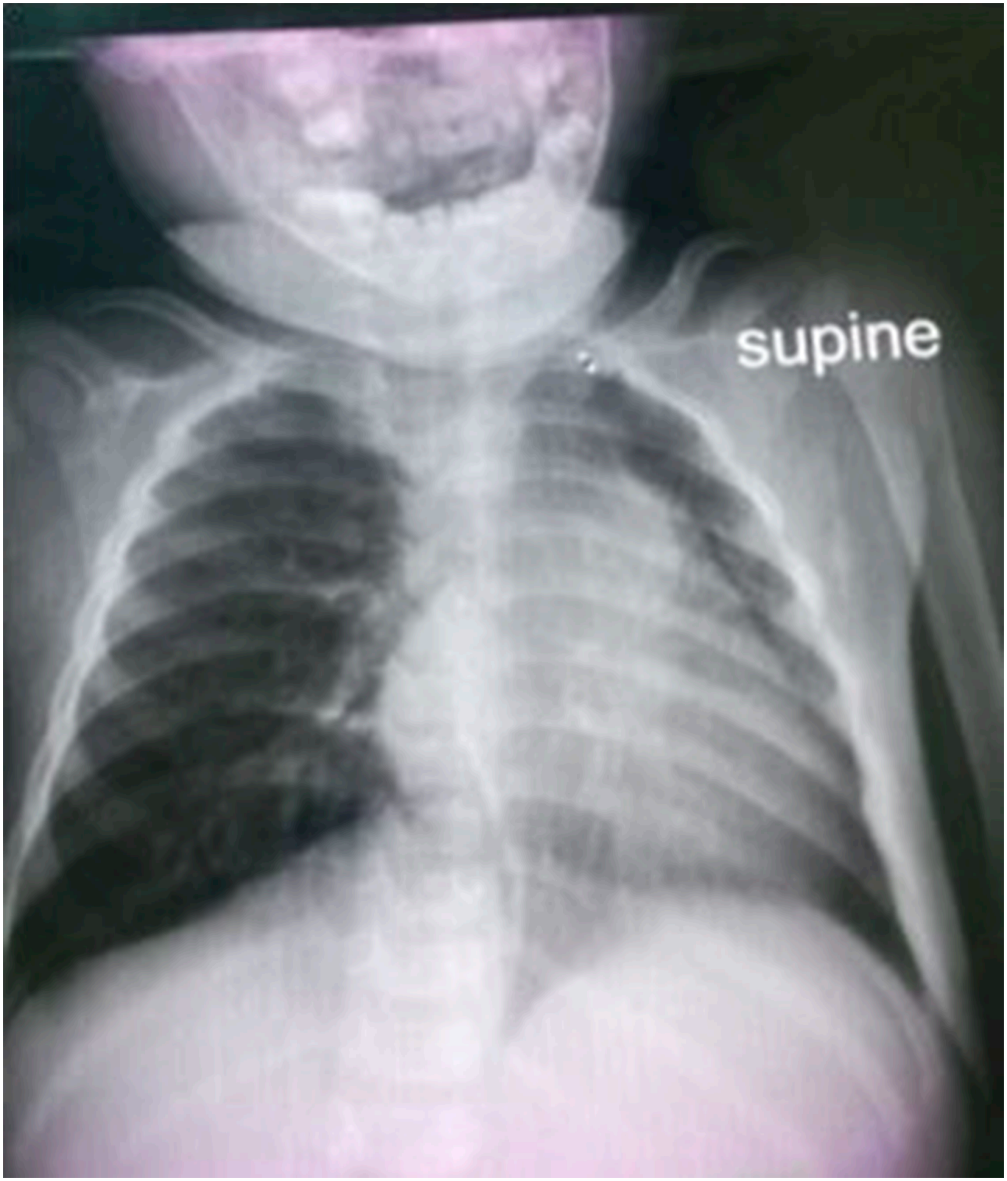
Chest X-ray of patient 2.

A 13-month-old boy presented with cough, drenching night sweats, and unexplained fever for 14 days. His father was diagnosed with RR-TB through sputum 5 days prior to this presentation. CXR showed left airway compression suggestive of complicated lymph node enlargement (Figure 2). Stool sample was collected on spot and was MTB detected high and RR. Nine-month regimen was initiated with delamanid (2Dlm-Lfx-Lzd-Cfz-Cs/4Dlm-Lfx-Cfx-Cs/3Lfx-Cfx-Cs).

### Patient 3

**FIGURE 3.**
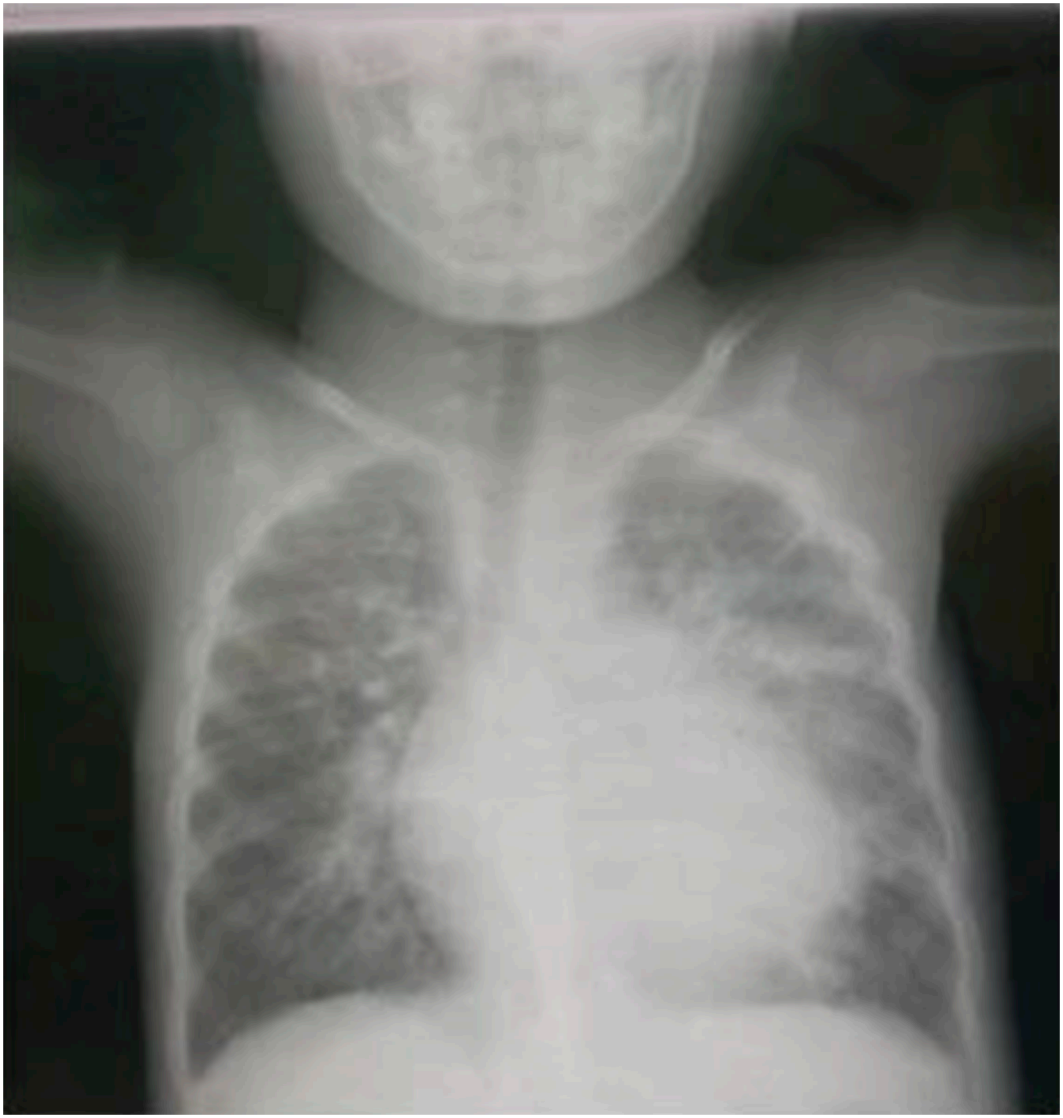
Chest X-ray of patient 3.

A 6-year-old girl presented with persistent cough, poor weight gain, reduced playfulness, and unexplained fever for 30 days. She had no contact of TB. On examination, she had fever and CXR had bilateral perihilar infiltrations (Figure 3). Stool sample came MTB detected low and RR. Nine-month regimen was initiated with bedaquiline (2Bdq-Lfx-Lzd-Cfz-Cs/4Bdq-Lfx-Cfx-Cs/3Lfx-Cfx-Cs).

### Patient 4

**FIGURE 4.**
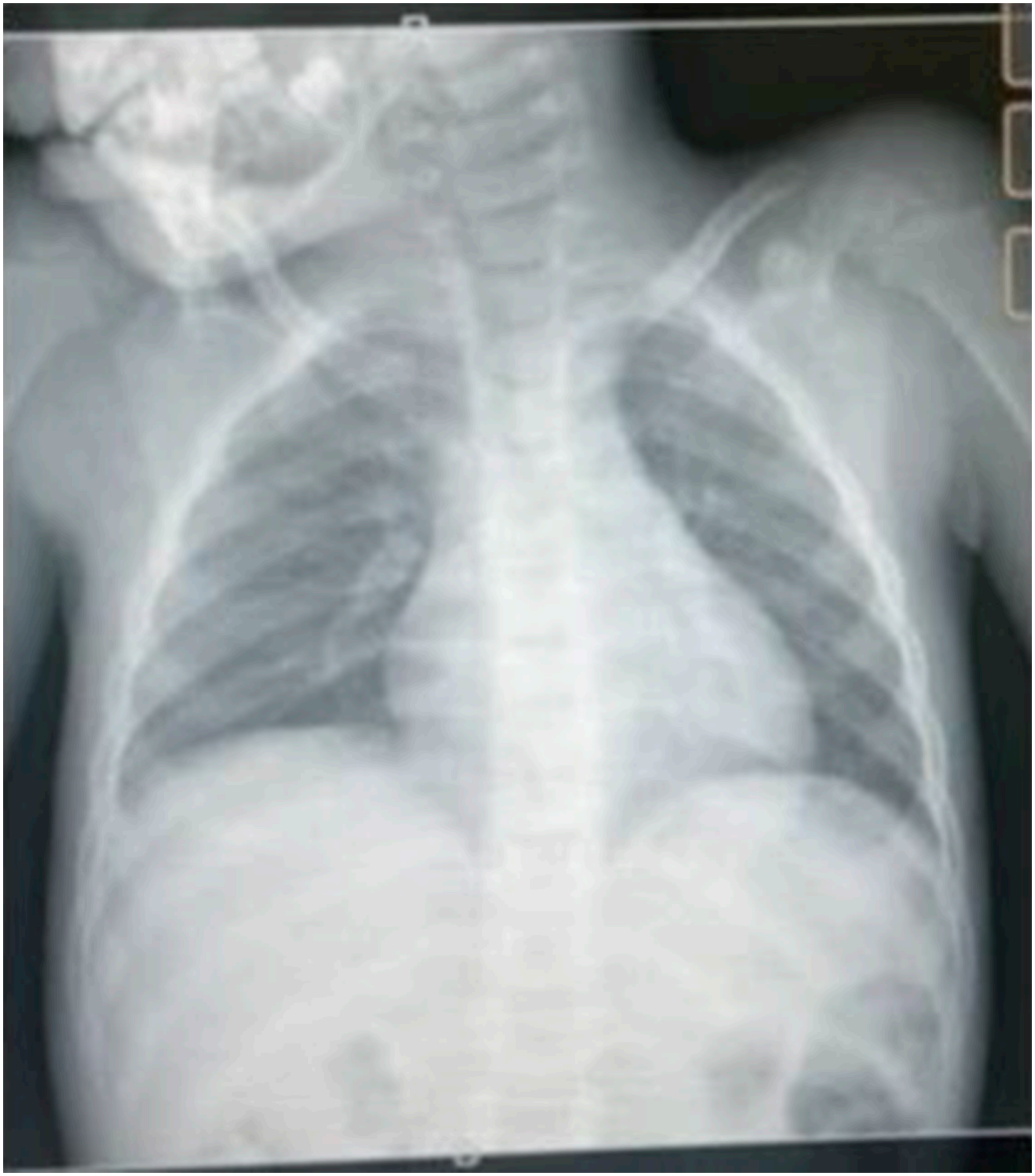
Chest X-ray of patient 4.

A 4-year-old girl presented with cough, poor weight gain, reduced playfulness, and unexplained fever for 14 days. She had a contact history of TB (uncle who died while on TB treatment in 2022). On examination, she looked sick with lymph nodes which were asymmetrical, painless, and localised. CXR showed enlarged right hilar (Figure 4). Stool sample^12^ came MTB detected medium with RR. Nine-month regimen was initiated with delamanid (2Dlm-Lfx-Lzd-Cfz-Cs/4Dlm-Lfx-Cfx-Cs/3Lfx-Cfx-Cs).

## DISCUSSION

Our study illustrates various clinical presentations of RR-TB in children and the important role that stool Xpert played in making the diagnosis of the four children. They received Bacillus Calmette-Guérin after birth, and all were HIV negative. They had moderate malnutrition as a risk factor for TB, and one patient was a direct contact of RR-TB.^13^ Children are often not able to produce sputum on their own, and in the circumstances where sputum is available, bacteriological diagnosis and confirmation may be unreliable as the sputum may contain very few or undetectable levels of the bacteria.^14^ In the absence of known contacts with RR-TB, three of these children would likely have been misdiagnosed in the absence of bacteriologic confirmation. CXR can support the clinical diagnosis of pulmonary TB when TB is presumed and bacteriological testing is negative.^15,16^ In our case, all the patients had features suggestive of TB on CXR.

All the patients failed to produce sputum due to their age and the other option was to collect stool sample. As recommended by WHO,^17^ stool samples were collected on spot and guardians were given a container with instructions on how to collect the stool sample.^8,12,18^ For patients 1, 3, and 4, without a sample, they would not have been suspected of RR-TB without a contact history and given appropriate treatment. We might have thought they had TB but not RR-TB, and patient 1 would have needed lymph node aspirate, which is invasive sampling, and even referral to a higher level of care, which would have also introduced logistical and financial burdens for the families. Patient 2 had RR-TB contact, and with that, some providers might have empirically started him on RR-TB treatment while others would have started him on drug-susceptible TB treatment, which could have led to complications in this child age, including meningitis. In this case, it was good to confirm diagnosis of RR-TB in this child. Turnaround time was rapid as all the children had results within 24 h and they were successfully treated under a Directly Observed Treatment programme, as it is reported that when diagnosed and started on treatment, children tend to do well.^19^

## CONCLUSION

Our four patient scenarios show the clinical benefits of stool sampling. While the stool method has been validated and recommended by the WHO, there have been limited reports about how it impacts the clinical management of children, especially in high-burden settings. These children would likely not have been offered RR-TB treatment without bacteriological diagnosis. A lack of treatment would have resulted in high morbidity and mortality.

## References

[1] Reuter A, Preventing tuberculosis in children: a global health emergency. Paediatr Respir Rev. 2020;36:44-51. 32253128 10.1016/j.prrv.2020.02.004

[2] Gebre M, Variable diagnostic performance of stool Xpert in pediatric tuberculosis: a systematic review and meta-analysis. Open Forum Infect Dis. 2021;8(8):ofaa. 10.1093/ofid/ofaa627 PMC8378590 34430668

[3] World Health Organization. Global tuberculosis report 2023. Geneva: WHO, 2023.

[4] World Health Organization. WHO consolidated guidelines on tuberculosis. Module 3: diagnosis - rapid diagnostics for tuberculosis detection, 2021 update. Geneva: WHO, 2021. 34314130

[5] Jenkins HE, Yuen CM. The burden of multidrug-resistant tuberculosis in children. Int J Tuberc Lung Dis. 2018;22(5):3-6. 10.5588/ijtld.17.0357 PMC5975247 29665947

[6] World Health Organization. WHO consolidated guidelines on tuberculosis: module 5: management of tuberculosis in children and adolescents, 2022. Geneva: WHO, 2022. 35404556

[7] World Health Organization. WHO operational guidelines on tuberculosis: module 5: management of tuberculosis in children and adolescents, 2022. Geneva: WHO, 2022. 35404556

[8] Yenew B, Diagnostic accuracy, feasibility and acceptability of stool-based testing for childhood tuberculosis. ERJ Open Res. 2024;10(3):00710-02023. 38770005 10.1183/23120541.00710-2023 PMC11103712

[9] Harichander S, New advances in pediatric drug-resistant tuberculosis. PAMJ One Health. 2025;16:11.

[10] World Health Organization. WHO global lists of high burden countries for tuberculosis (TB), TB/HIV and multidrug/rifampicin-resistant TB (MDR/RR-TB), 2021–2025: background document. Geneva: WHO, 2021.

[11] Malawi national tuberculosis and leprosy elimination program. 9th ed. Lilongwe, Malawi: Ministry of Health, 2024.

[12] The SOS stool box: an implementation package for the SOS stool method to detect tb and rifampicin resistance. https://www.kncvtbc.org/en/sos-stoolbox/.

[13] Akalu TY, Prevalence of tuberculosis infection among contacts of drug-resistant tuberculosis patients: a systematic review and meta-analysis. J Infect. 2024;89(2):106198. 38906264 10.1016/j.jinf.2024.106198

[14] Soriano-Arandes A, Clinical presentations and outcomes related to tuberculosis in children younger than 2 years of age in Catalonia. Front Pediatr. 2019;7:238. 31245340 10.3389/fped.2019.00238 PMC6579838

[15] World Health Organization. WHO consolidated guidelines on tuberculosis module 5: management of tuberculosis in children and adolescents. Geneva: WHO, 2022. 35404556

[16] Vonasek B Screening tests for active pulmonary tuberculosis in children. Cochrane Database Syst Rev, 2021;6(6):CD013693. 34180536 10.1002/14651858.CD013693.pub2 PMC8237391

[17] World Health Organization. Rapid communication on updated guidance on the management of tuberculosis in children and adolescents. Geneva: WHO, 2021.

[18] De Haas P, The simple one step (SOS) stool processing method for use with the Xpert MTB/RIF assay for a child friendly diagnosis of tuberculosis closer to the point of care. J Clin Microbiol. 2021;59(8):e00406-e00421. 34076469 10.1128/JCM.00406-21 PMC8373220

[19] Garcia-Prats AJ, Characteristics of children and adolescents with multidrug-resistant and rifampicin-resistant tuberculosis and their association with treatment outcomes: a systematic review and individual participant data meta-analysis. Lancet Child Adolesc Health. 2025;9(2):100-111. 39855750 10.1016/S2352-4642(24)00330-4 PMC13289721

